# Impact of Calcium Oxide on Hygienization and Self-Heating Prevention of Biologically Contaminated Polymer Materials

**DOI:** 10.3390/ma13184012

**Published:** 2020-09-10

**Authors:** Katarzyna Wolny-Koładka, Mateusz Malinowski, Witold Żukowski

**Affiliations:** 1 Department of Microbiology and Biomonitoring, Faculty of Agriculture and Economics, University of Agriculture in Krakow, al. Mickiewicza 24/28, 30-059 Krakow, Poland; 2 Department of Bioprocesses Engineering, Energetics and Automatization. Faculty of Production and Power Engineering, University of Agriculture in Krakow, ul. Balicka 116b, 30-149 Krakow, Poland; mateusz.malinowski@urk.edu.pl; 3 Faculty of Chemical Engineering and Technology, Cracow University of Technology, Warszawska 24, 31-155 Krakow, Poland; witold.zukowski@pk.edu.pl

**Keywords:** spent polymer materials, alternative fuel, microorganisms, calcium oxide (CaO)

## Abstract

During the storage of spent polymer materials derived from municipal solid waste, which contain biodegradable impurities, an intense growth of microorganisms takes place. The aerobic metabolism of microorganisms may cause these materials to combust spontaneously and to become a real epidemiological risk for humans. The aim of the research is to determine the optimal addition of calcium oxide (CaO), which effectively reduces the number of selected microorganism groups populating the analyzed materials, in which spent polymers represent a significant fraction: refuse-derived fuel (RDF) and an undersize fraction of municipal solid waste (UFMSW). The main novelty of the experiments is to assess the benefits of using the commonly available and cheap filler (CaO), to hygienize the material and to reduce the fire hazard arising from its storage. During the mixing of spent polymer materials with pulverized CaO (mass shares: 1, 2, and 5% CaO), temperature changes were monitored using thermography. Moisture content (MC), pH, respiration activity (AT4) and bacterial count were determined before and after the experiment. During the addition of CaO (especially when the content was at 5%) to the UFMSW, higher maximum temperatures were obtained than in the case of RDF analyses, which may be the result of a high percentage of the biodegradable fraction and higher MC of the UFMSW. In all cases the waste temperature did not increase again after 3 min. CaO used in the experiment effectively limited the number of microorganisms. The addition of 5% of CaO has showed the strongest antimicrobial properties, and it can be recommended for hygienization of the analyzed materials and for the reduction of the risk of self-heating during their storage in windrows.

## 1. Introduction

The use of calcium oxide (CaO) is an extremely popular method of stabilization and hygienization of various spent materials, as it allows to deactivate and eliminate most microorganisms, including pathogens, endospores, viruses, and parasites [1]. Increase of pH induced by the addition of CaO causes permanent changes in ionization of the structural components of cellular proteins of microorganisms (especially in the case of anionic and carboxylic groups). This leads to irreversible modifications of their structures and disrupts the activity of the enzymes [2]. As suggested by Bień [3], the hydration of sewage sludge may reach as much as 99%. Owing to the addition of CaO, the share of organic compounds and the hydration of raw material are reduced, thus increasing the percentage of sludge dry matter; moreover, permanent binding of heavy metals occurs [4]. Hygienization using CaO is also used in composting of kitchen waste to eliminate odors from the process and to remove pathogenic microorganisms [5]. There is a lot of information on mixing CaO with refuse-derived fuel (RDF) in cement factories and on the impact of CaO on the rotted material [6] and the thermal processing of RDF [7]. As far as we know the source literature gives no information on the use of CaO for hygienization and prevention of spontaneous heating during the storage of polymer materials which can be obtained from the municipal solid waste (MSW) and are mainly used as refuse-derived fuels (RDF).

Biologically contaminated spent polymer fractions represent a real microbiological threat to humans and warehouses where such materials are stored [8]. According to Malinowski and Wolny-Koładka [9], Malinowski and Sikora [10] and Vasconcelos and Silva [11], the temperature of RDF stored in windrows may reach 75 ± 5 °C, whereas Hogland and Marques [12] and Yasuhara [13] suggest that the RDF temperature may reach 65 °C. The reason for the spontaneous heating of RDF is the decomposition of organic matter (present as contamination/impurities) by microorganisms [8]. Some studies describe cases of RDF explosions as a result of uncontrolled temperature increase [14], which poses a risk to the health of employees. Apart from the microorganisms, factors influencing the spontaneous heating of RDF are pressure in the windrows, degree of material fragmentation, size of windrows, and water content in the stored material [12].

Because of the possibility of spontaneous combustion [15] and its negative impact on the health of workers [16], entrepreneurs use various methods of protection and prevention of these harmful effects on the environment [17]. There are sensors, fire extinguishing systems, and infrared cameras installed in halls; materials undergo processes of ozonation [18], use of ultrasound or microwave radiation [19], biological stabilization [20], biological drying [21,22], or pH modifications. A popular method is also agglomeration of those materials [23,24] e.g., by the pellet production process [25,26] or granulation [27]. The most popular method is incineration in cement plants [28,29] or CHP (combined heat and power) plants [30].

Mixed MSW and the undersize fraction of MSW (UFMSW) produced from the mixed MSW with granulation below 80 mm are particularly harmful. This type of substrate is a habitat for microorganisms and a source of heat and odor emission [15,31]. UFMSW contains a relatively high percentage of organic substances (40–60%) [20]; therefore, it is directed for biological modification to reduce their volume and weight and subject to sanitation process [32]. RDF produced from the oversize fraction of MSW due to a high polymer content (>60%) [8] is mainly used in cement plants [21] as a coal substitute in the cement production process [31].

Self-heating of the RDF and the UFMSW results from the aerobic metabolism of microorganisms populating biodegradable (mainly organic) material fractions [33]. Malinowski and Sikora [10] stated, based on analyzing the morphological composition of RDF, that the percentage of organic fraction content may be >20%. Analyses carried out by Malinowski and Wolny-Koładka [8] showed that the share of the organic fraction in RDF may range from 11% to 29%.

Both the RDF and the UFMSW provide habitats for different groups of microorganisms, including pathogenic bacteria and toxigenic mold fungi [9]. The occurrence of microorganisms of the *Enterobacteriaceae* family in those materials (i.e., *E. coli*, *Salmonella* spp. and *Shigella* spp.), of the *Staphylococcus* genus, as well as bacteria generating endospores, i.e., *Clostridium perfringens* is a key problem [18]. A high concentration of these microorganisms, as well as their metabolites (endotoxins, enterotoxins, mycotoxins), may cause different diseases, such as respiratory tract inflammation, chemical or allergenic pneumonia, skin allergies, food poisoning, meningitis, and sepsis [34]. The presence of pathogenic microorganisms in wastes is not only a potential epidemiological threat, but it may also cause microbiological contamination of the environment [18].

All of the above-mentioned properties of UFMSW and RDF lead to the rapid temperature increase in the stored waste (usually heaps piled in the shape of triangular prisms (windrows)). The temperature increase in windrows was also investigated in these studies.

This study is to show that the addition of CaO to the RDF and UFMSW effectively leads to hygienization of such material and reduces its ability to self-heating capacity. The innovative character of the suggested solution consists in using the commonly available and cheap filler (CaO) as a structural additive to the analyzed materials. The use of CaO may result in reducing the fire hazard posed by its storage. The analysis was conducted to recommend practical conditions (CaO addition) for industrial production of RDF and UFMSW (with different moisture content). The scope of the study includes microbiological analyses of materials before and after the addition of CaO, thermographic evaluation of temperature inside the windrows of UFMSW and RDF (on different depth) and thermographic evaluation of the process of mixing waste with the filler, and the analysis of the impact of CaO addition on the moisture content (MC), pH and the waste respiration activity (AT4). Three different CaO percentages were used in the test (1, 2 and 5% by weight) as well as RDF and UFMSW of different MC.

## 2. Materials and Methods

### 2.1. Materials

The tested materials were obtained from the MIKI Recycling Ltd. (Krakow, Poland) RDF production installation (50.032445247N, 20.061035156E). UFMSW and RDF, products of MSW mechanical treatment, were analyzed. During MSW mechanical treatment, waste is directed to preliminary shredding (tearing the sacs) and then to magnetic and manual separation which results in retrieving recyclable materials: ferrous and nonferrous metals, glass, paper, and cardboard. The remaining waste stream is directed to a trommel screen with an 80 mm mesh, where waste is separated into fine fraction of 0–80 mm grain size (UFMSW) and into thick fraction over 80 mm [20,32]. A fraction over 80 mm grain size is directed to an air separation unit where further separation into heavy and light fractions is performed. The light fraction (pre-RDF) that contains mostly spent polymer materials is directed to final shredding. RDF prepared in such a way is immediately loaded onto vehicles and shipped to a cement factory, because of self-ignition risk [25,31].

The shares of polymers in the tested materials calculated on the basis of [8,10] were 65% and 45% for RDF and UFMSW, respectively. Because of a varying MC in the UFMSW and the RDF, materials with the following MC were used in the study (±2%):

- UFMSW: 35, 50 and 60% (control sample: 35%);
- RDF: 20 and 30% (control sample: 20%).

CaO is a strong disinfectant that generates heat (high temperature) during the exothermic reaction with water [33]. To meet the research objective, pulverized, highly reactive (t_60_ < 1.5 min) CaO, type EN 459 CL 90-Q (manufacturer: Lhoist Ltd., Bukowa, Poland) was used.

### 2.2. Sampling and Experiment

Sampling at the MIKI Recykling Ltd. (Krakow, Poland) and laboratory tests were carried out in three replications for each variation (addition of CaO and different moisture contents of tested materials). Test samples were taken in accordance with the method recommended by the European Committee for Standardization, 2006, Characterization of Waste—Sampling of Waste Materials—Framework for the Preparation and Application of a Sampling Plan [35]. More than 30 unit samples were taken for each replication. The RDF collected for testing is stored in the hall in the form of irregular piles, dimensions: base 6 m × 10 m and height up to 3 m. The UFMSW collected for experiments is stored in the form of regular piles, dimension: base 10 m × 50 m and height up to 4 m.

The samples were placed in containers and immediately transported to the laboratory for laboratory tests. The materials delivered to the laboratory were divided into representative samples with weights from 1000 to 3000 g. MC, pH, AT4, and the number of microorganisms were determined in the samples. Control samples were subjected to a thorough physical, chemical, and microbiological analysis in order to characterize the studied materials precisely.

The experiments consisted in the application of CaO in the hygienization process. To reduce the capacity of tested materials for self-heating, the heat generated as a result of hydration was used. Moreover, as a result of the reaction of CaO and H_2_O, OH^−^ ions are released; they are strongly alkaline and highly toxic to microorganisms. Hygienization using the CaO addition followed the scheme of exothermic reaction as described by the following Equation (1):

CaO + H_2_O → Ca(OH)_2_ + 1140 kJ∙kg^−1^CaO (1)

The tested material (RDF or UFMSW) with weight 5000 g and various initial moisture content was placed in the mixer with 80 dm^3^ capacity, completing 22–24 rotations per minute. CaO was added in three different weight fractions: 1%, 2%, and 5%, respectively. All experiments were performed in triplicate (for different initial MC and CaO addition). The duration of the mixing process was 10 min. During that process, the temperature of the mixture was measured in 15 s intervals using the thermographic camera (ThermaCAM e300, FLIR, Warszawa, Poland). The initial temperature of materials used in the study was 16.6 ± 1.3 °C, whereas the temperature in the laboratory was 18.1 ± 1.1 °C.

### 2.3. Thermographic Analysis

Thermography is a method of recording infrared radiation using the thermographic camera [21]. Thermal radiation (wavelength range from 0.9 to 14 μm) is detected by the optical system of the camera and converted in the detector into an electrical signal [33]. Owing to the thermographic camera (ThermaCAM e300, FLIR, Warszawa, Poland), non-contact measurement of the temperature of the studied object is possible, with the temperature resolution of 0.1 °C. Each recorded thermogram has 16-bit color depth and the size of 320 × 240 pixels, so the data matrices provide information on the temperature of 76,800 points. The thermogram presents only the image of the apparent temperature; therefore, the emissivity coefficient (=0.95) of the tested materials calculated in previous studies had to be taken into account during the analysis [8]. The determination of the emissivity was performed based on the black tape methodology, using an Iso Tape Tesa adhesive tape (material with a known emissivity value of 0.97 at 35 °C) (TESA, Hamburg, Germany) A piece of adhesive tape was glued on the material, and then evenly heated the object to a temperature of 35 °C. The temperature was registered by a thermographic camera set to the emissivity coefficient of the adhesive tape. We aimed the camera at an area without the tape and changed the emissivity coefficient of the camera until the temperature of the tested material was the same as the previously measured temperature of the tape. The emissivity was determined separately for each material that was part of RDF and UFMSW. The result was averaged according to the morphological composition.

The thermographic evaluation was used in the study for two purposes. By using thermography, the temperature inside the stored materials at various depths was intended (24 h after they were manufactured). The studies lasted for 12 months in MIKI Recycling Ltd. (Krakow, Poland). The methodology for making this measurement using a thermographic camera was described in detail in Malinowski and Wolny-Koładka [8]. In addition, an infrared camera was used in the temperature measurement carried out through the feeding hole of the device during a momentary stoppage of the mixer (mixing materials with CaO in different doses). In the QuickReport 2.1 software (FLIR, Wilsonville, OR, USA), the thermogram was corrected, as the following parameters had to be taken into account: the distance between the camera and the material (0.75 m—photo taken from the camera stand), ambient temperature and relative air humidity (45%). Subsequently, maximum, average, and minimum temperature values were read for each thermogram. Those temperature readings allowed us to prepare a graphical interpretation of temperature dynamics.

### 2.4. Microbiological, Physical, and Chemical Analyses

All samples (15) subjected to the experiment were subsequently analyzed in the laboratory. Using the pH-meter (Emerton, Poland), pH values of all the tested materials were examined. MC was determined in accordance with CEN-TS 15414-1: 2006 Solid Recovered Fuels [36]. It was done using the oven-dry method. Samples weighing approximately 300 g were distributed on the tray and weighed with accuracy to 0.1 g. The sample was placed in the laboratory dryer heated to 105 ± 2 °C. After 60 min, the test tray was weighed again. The procedure was repeated until the sample mass did not exceed 0.2% of the previous weight. On that basis, materials with specific MC levels (±2%) were selected for further analyses. This test was carried out in three replications for control samples and all mixtures with CaO.

AT4 was determined using the OxiTop system (WTW, Wrocław, Poland) according to WTW/OxiTop [37]. This method consists in placing 40 g of sample in a sealed container with a volume of 2.5 dm^3^ equipped with a pressure sensor. Inside, there was a vessel with a carbon dioxide (CO_2_) absorbing compound. The vacuum value in the container was read by the controller for 5 days. The vacuum value was converted into the amount of oxygen consumed during the process in the four-day period (AT4), according to the transformed formula of Clapeyron [38]. The AT4 analysis was carried out in three replications for the samples before and after the addition of CaO, to determine respiratory activity changes in the examined materials.

In order to isolate microorganisms, 10 g of the tested material was weighed from each sample. The isolation was carried out using the micro-dilution method according to the Koch method [9], using microbiological substrates. Table 1 shows the groups of microorganisms, the names of the nutrients used in the tests, and the time and temperature of incubation.

The numbers of vegetative and spores bacteria point to an abundance of nutrients readily digestible for microorganisms in the studied raw materials. The high numbers of bacteria, mold fungi, and actinobacteria also suggest the occurrence of favorable conditions (temperature, substrate pH, MC) for the growth of microorganisms [18]. The occurrence of potentially pathogenic bacteria was studied too, i.e., the presence of *Staphylococcus* spp., *Escherichia coli*, *Salmonella* spp. and *Shigella* spp., *Enterococcus faecalis*, *Clostridium perfringens*. The analysis of micro-dilutions was carried out in three replications. The number of colony-forming units (CFU) of microorganisms was determined by calculating the result of identification per one gram of the dry sample mass (CFU·g^−1^ d.m.). For initial identification of microorganisms isolated from the samples, bacteriological preparations were made using the Gram staining method, as well as vital preparations in the iodine solution [9,18].

The statistical analysis of the results was performed using the Statistica software v. 12.5 (StatSoft, StatSoft Polska, Kraków, Poland). The variance analysis was conducted to verify the relevance of MC, pH, AT4 and the number of selected groups of microorganisms in waste depending on the fraction tested and the percentage of CaO added.

## 3. Results

### 3.1. Characteristics of RDF and UFMSW

RDF produced from MSW should possess specific properties, usually determined by recipients of alternative fuel (cement plants) [39]. The most important properties include chlorine content, MC, and calorific value [40]. UFMSW usually is characterized by low heavy metal content which determines it being deposited in landfills after biological treatment processes [20]. Table 2 and Table 3 present physicochemical and microbiological characteristics of analyzed materials. Parameters of RDF and UFMSW did not deviate from those discussed in the source literature [9,32]. The content of heavy metals in the control samples was relatively low.

Table 3 shows that microorganisms in UFMSW are more abundant than those in RDF. It results from a higher organic fraction content in the UFMSW, which provides a breeding ground for microorganisms. Their number is similar to the values presented in other studies [9,18]. Results concerning the number of actinobacteria and the *Shigella* spp. are not included, as those microorganisms were not found in the studied raw materials.

CaO used in the study was completely free of microorganisms, which directly resulted from a very high pH. Highly reactive CaO had CaO content of >96%, MgO < 0.5%, SO_3_ ≈ 0.1%, CO_2_ ≈ 1.1%, and reactivity of t_60_ = 25 sec. The density of the pulverized CaO used in the study was 0.8 kg·m^−3^.

### 3.2. The Temperature of Materials Stored in the Windrows

**Figure 1:**
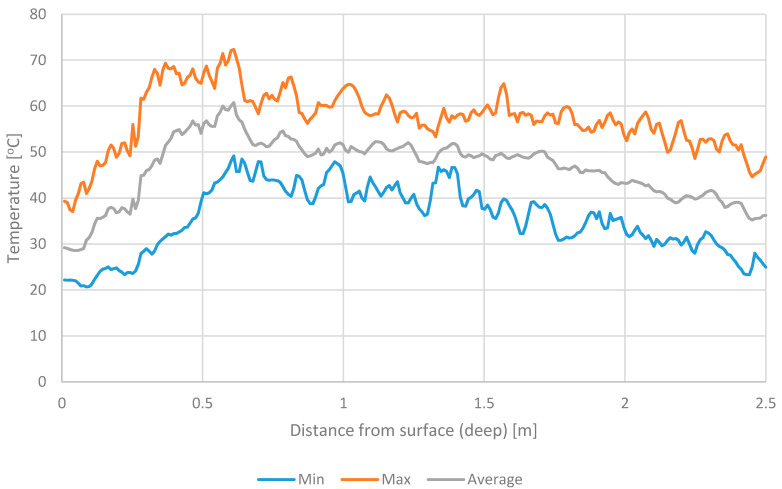
The temperature of refuse-derived fuel (RDF) at different depths.

**Figure 2:**
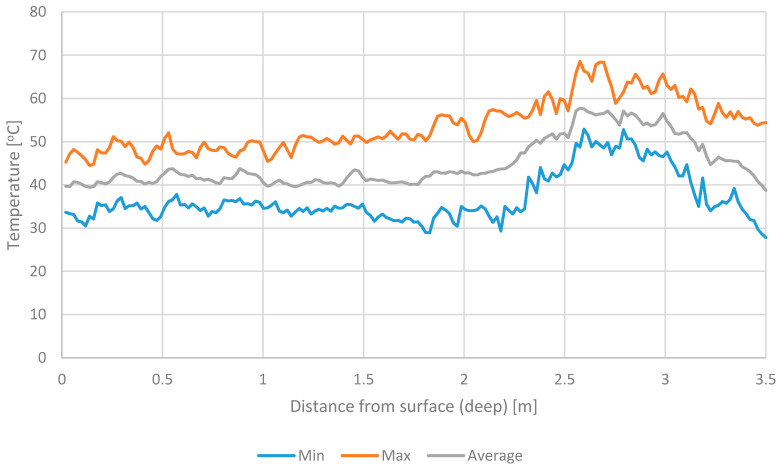
The temperature of an undersize fraction of municipal solid waste (UFMSW) at different depths.

Based on the previous studies [8,33] it was found that both RDF and UFMSW heat up within 24 h from their manufacturing. Figure 1 and Figure 2 show the temperature of waste at different depths after 24 h of their storage. Test results illustrate maximum, minimum, and averaged temperature data for 12 months. For both RDF and UFMSW, a temperature increase up to 70 °C was observed at different depths, regardless of the month of the year. The main difference was that the warmest place of RDF occurred at a depth of 0.3–0.6 m below the windrow surface, while in the case of UFMSW, it was between 2.5 and 3 m. High temperatures demonstrated in the thermographic analysis, confirmed the need to limit the temperature increase in the stored RDF and UFMSW. In the case of RDF, it is important because of the possibility of self-ignition, which is closely related to the time of storage in windrow [10,11,12,13]. In the case of UFMSW, long-term storage may pose a threat to the health of employees (odor emission, increase in the number of pathogenic microorganisms) [18,34].

### 3.3. Impact of CaO Addition on the Temperature of the Analyzed Materials during the Mixing

**Figure 3:**
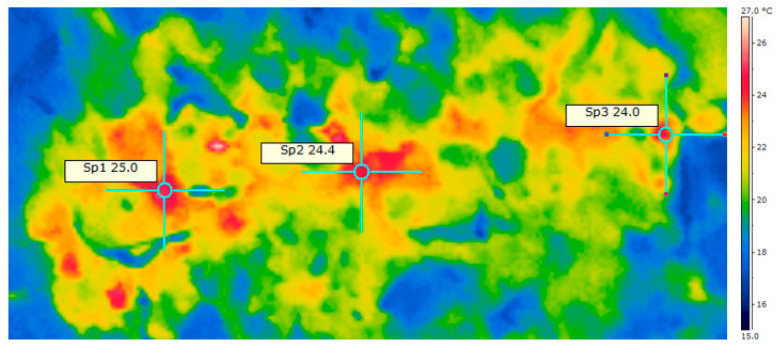
Thermograph from QuickReport 2.1 software with a view of materials in the chamber during mixing with CaO (RDF + 5% mass addition of CaO).

**Figure 4:**
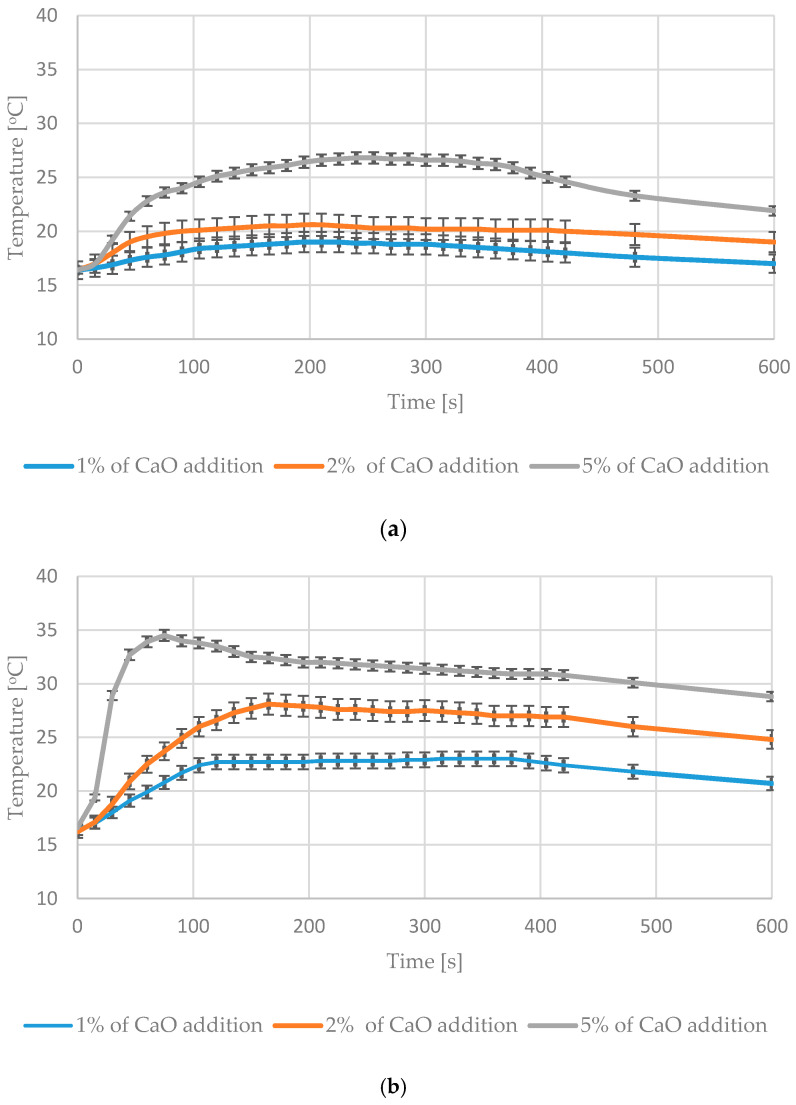
Temperature changes during mixing of materials: (**a**) RDF with 20% moisture content and different mass additions of CaO, (**b**) RDF with 30% moisture content and different mass additions of CaO.

**Figure 5:**
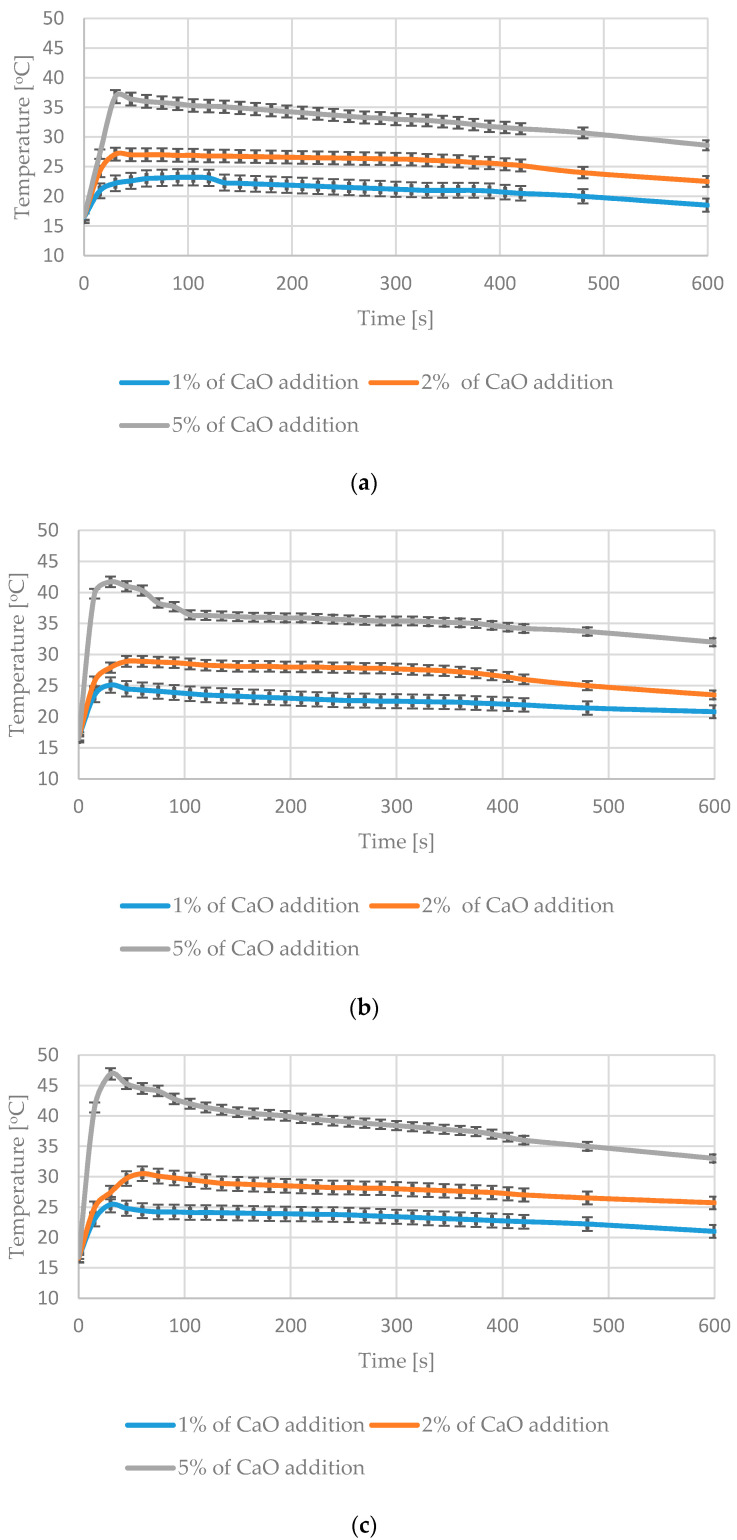
Temperature changes during mixing of materials: (**a**) UFMSW with 35% moisture content and different mass additions of CaO, (**b**) UFMSW with 50% moisture content and different mass additions of CaO, (**c**) UFMSW with 60% moisture content and different mass additions of CaO.

Figure 3 presents an exemplary thermographic photo made for RDF mixed with the 5% CaO addition. For each picture in QuickReport 2.1, an average temperature of waste visible through the mixer feeding hole was read. It allowed us to show temperature changes of tested materials in time (Figure 4 and Figure 5).

Adding CaO to RDF caused the temperature increase of materials placed in the mixer. The increase was visibly higher in the case of RDF (with 30% moisture content). For the 5% CaO addition to the RDF with the 30% moisture content, the temperature of waste increased to 34 °C within 60 s after adding CaO (Figure 4).

During the mixing of the UFMSW with CaO, temperature maxima were significantly higher in comparison with the RDF case. It was due to higher moisture content and a high percentage of biodegradable fraction in UFMSW [10,20]. The high temperature during the mixing process (exceeding 40 °C) was observed only in UFMSW with the MC exceeding 50% after adding 5% CaO. The reaction was relatively short—no more than several minutes. Maximum temperatures were reached within 2–3 min after adding CaO, both for RDF and UFMSW. The temperature was rising most dynamically in the first 2 min of the mixing process (Figure 5). The added percentage of CaO determined the heating rate of the sample. Temperature maxima differed from sample to sample, depending on the initial MC. Standard deviations marked in Figure 4 and Figure 5 are the lowest with the 5% addition of CaO. Significant differences within one test series (e.g., with 1% CaO addition) result in our opinion from the heterogeneous composition of RDF and UFMSW. After the mixing process, the temperature stopped increasing. Both materials were initially stabilized and their capacity of self-heating was successfully reduced.

### 3.4. Impact of CaO Addition on Moisture Content, pH, and Respiration Activity (AT4)

Reduction of MC in the tested samples after the addition of CaO and thorough mixing, which was statistically significant, was observed only in the case of 5% CaO addition, and only in the UFMSW samples with the initial moisture contents of 50% and 60%. The moisture content of both materials decreased by 11.8%. In the remaining cases, no statistically significant differences in the MC of samples were found (Table 4). It is related to the dynamics of temperature changes during mixing (correlation coefficient R = 0.9). The moisture content of the materials decreased significantly only in those cases where the process temperature was over 40 °C.

CaO additions contributed to a significant pH increase in the tested materials, up to 12.35 for RDF and 12.48 for UFMSW (RDF control sample: 8.31 and UFMSW control sample: 7.54). The highest pH increase (over 61%) was observed for UFMSW with the initial MC: 50 and 60%, regardless of the CaO addition (correlation coefficient R = 0.75). The pH changed from neutral, which is optimal or acceptable for most microorganisms, to strongly alkaline, which should inhibit the growth of most microorganisms.

The addition of CaO did not influence the respiration activity of RDF in a statistically significant manner, however, in the case of 2% and 5% CaO additions to UFMSW the average value of AT4 decreased by 2.3 and 7.4 mgO_2_∙g^−1^d.m, respectively. The AT4 decrease was significantly associated with a decrease in moisture contents (R = −0.8). Vital processes in those materials were reduced, which proves that they had been initially stabilized.

### 3.5. Influence of CaO Addition on the Number of Microorganisms

The microbiological analyses allowed to assess changes in the numbers of studied microorganisms both in the RDF and UFMSW samples subjected to the process (Table 5). The 5% CaO addition had the highest impact on reducing of the number of vegetative bacteria in both tested materials (from 350,000 × 10^2^ CFU·g^−1^ d.m. in the control sample of RDF to 794 × 10^2^ CFU·g^−1^ d.m., and from 636,418 × 10^2^ CFU·g^−1^ d.m. in the control sample of UFMSW to 1991 × 10^2^ CFU·g^−1^ d.m.). The number of spores bacteria also decreased as a result of the addition of CaO in comparison to the control samples (from 1567 × 10^2^ CFU·g^−1^ d.m. in RDF to 58 × 10^2^ CFU·g^−1^ d.m., and from 5207 × 10^2^ CFU·g^−1^ d.m. in UFMSW to 108 × 10^2^ CFU·g^−1^ d.m.). In one of the UMFSW samples (with the moisture content of 50%), however, the reduction of the number of spores bacteria was less spectacular (to 4012 × 10^2^ CFU·g^−1^ d.m.). The reason might have been that the spores bacteria produce endospores, which allow them to survive in unfavorable conditions. Moreover, the structure of waste is irregular, and its porosity is high—these factors undoubtedly facilitate colonization of the waste surface by microorganisms. The high moisture content (50%) in the analyzed sample was also significant—it might have influenced the growth of spores bacteria. The hygienizing effect of CaO addition was also observed in mold fungi. Their number decreased in all samples compared to the control sample. For two RDF samples (with moisture contents of 20 and 30%, respectively) with the addition of 5% CaO, and for one UFMSW sample (with the moisture content of 60%) with the addition of 1% CaO increased numbers of mold fungi were found as compared with the control sample (22,876, 5637, and 11,539 × 10^2^ CFU·g^−1^ d.m., respectively).

The number of bacteria of the *Staphylococcus* genus varied enormously from sample to sample. In the case of RDF, the result of the CaO addition was inconclusive, as a decrease in numbers of staphylococci was observed in the samples with 2% CaO addition (1950 and 1688 × 10^2^ CFU·g^−1^ d.m.), whereas in the samples with 5% addition, the number of staphylococci were either radically reduced (1065 × 10^2^ CFU·g^−1^ d.m.) or increased (16,427 × 10^2^ CFU·g^−1^ d.m.) depending on the MC in the analyzed sample in comparison with the control sample (17,380 × 10^2^ CFU·g^−1^ d.m.). In the case of UFMSW, the 1% CaO addition had the least waste hygienization effect, whereas 2% and 5% additions effectively reduced the number of staphylococci (max. up to 1497 and 1114 × 10^2^ CFU·g^−1^ d.m., respectively) compared to the control sample (17,355 × 10^2^ CFU·g^−1^ d.m.). The number of the other pathogenic bacteria, i.e., *E. coli*, *Salmonella* spp., *E. faecalis,* and *C. perfringens* visibly decreased, and in some cases down to zero. Regardless of the moisture content in the raw materials, the CaO addition, particularly that of 5%, had a strongly hygienizing influence and contributed to the microbiological stabilization of waste.

## 4. Discussion

RDF and UFMSW vary enormously in terms of organic matter content which constitutes a source of nutrients for microorganisms [18]. Also, the structure of the tested materials is irregular, its morphology is heterogeneous, and their porosity is high; these factors undoubtedly facilitate the colonization of the waste surface by microorganisms [41], but the polymer fraction in waste always consists of materials of very similar chemical properties, including those causing flammability and the possibility of ignition [21]. This is due to the mass production of plastics around the world and the fact that they are always thermoplastic polymers with the same or very similar physicochemical characteristics (PE, HDPE, LDPE, LLDPE, PP, PS, PET etc.). Also, the paper fraction in waste, regardless of the sample, will have similar properties as regards the possibility of biological decomposition and flammability. The waste is inhabited by mixed populations of microorganisms characterized by high biodiversity, which means that it is very difficult to deactivate them effectively [42,43]. Single-celled organisms living on this material will be the same or very similar. Metabolic mechanisms, namely the mechanisms of aerobic respiration responsible for the rapid release of heat will be the same, which is due to the universality of simple organisms in nature and the similarity of their major metabolic pathways [18].

Considering a mixed character of the microorganism population settling the RDF and UFMSW, a higher temperature adjusted to various microorganism groups with a wide tolerance range to extreme factors should be applied for a proper hygienization [44,45]. An inexpensive, fast, and simple method, whose efficiency was confirmed by the tests described above, is the addition of highly reactive CaO (in small doses) to the waste (RDF and UFMSW). Increased temperature and strongly alkaline pH caused a statistically significant reduction in the number of microorganisms [33]. Hygienization of waste caused by CaO addition also allowed to prevent waste from self-heating. The tested materials have lost the ability to heat spontaneously. The method proposed in the article works in each case on all microorganisms and does not depend on the variable components of the waste.

Gajewska et al. [21] suggest that the using of the process of biological drying may prevent the spontaneous heating of waste. Biodrying requires substantial expenditure (specialized infrastructure), and the treatment takes several days. Jewiarz et al. [25] point out that RDF palletization as a simple mechanical process will have a similar effect to those presented in this paper; at the same time, they underline the necessity of drying the spent polymeric materials prior to the process, which requires considerable energy expenditure, and, in consequence, considerable economic efforts. CaO is added to the waste mostly in the stage of their thermal treatment in cement factories [7]; but to eliminate the risk of self-heating, CaO may be added before they are transported to treatment plants. Tatemoto et al. [6] noted that the addition of CaO before the RDF production process is effective for suppression of rots and eliminates *E. coli*, similarly to the tests described in this paper (Table 5). According to the authors, however adding CaO to the waste at such an early stage may have adverse effects due to the lacking control of CaO content in materials after their further mechanical treatment.

Famielec et al. [33] analyzed the impact of CaO on mixed MSW with initial moisture contents of 35% and 40%. Results of their work are consistent with those obtained in analyses performed for UFMSW. Similarly, to Famielec et al. [33], the duration of CaO reaction with the waste is short—not exceeding several minutes. In contrast to this research [33], it was not found that the initial moisture content of the samples influenced the temperatures achieved during the process in all cases. Maximum temperatures were reached within 2 min after adding CaO, much faster than in the studies by Famielec et al. [33]. In the research of Wong and Fang [46], the waste (sewage sludge) temperature was higher (over 50 °C even with the CaO addition below 1%). Wong and Fang [46] state that CaO has adverse effects on all biological parameters, increasing with the increasing CaO application rates, but these effects are generally confined to the early stage of waste treatment.

## 5. Conclusions

Biological contaminated spent polymer materials (RDF and UFMSW) are a mix of different material types, including organic ones (green and kitchen waste, paper, cardboard, etc.), which are perfect breeding grounds for microorganisms. The analysis of the microbiocenotic composition of RDF and UFMSW samples showed that waste hygienization with the small doses of CaO delivers very good results. The proposed process contributed to reduce significantly numbers of most microorganisms in the tested raw materials, including pathogenic microorganisms. The best hygienization effects were recorded for the 5% CaO addition. In addition, the highest temperature increased and reduction of MC after the addition of CaO and thorough mixing was observed in the case of 5% CaO addition in the UFMSW samples with the initial moisture contents of 50% and 60%.

The main benefit of using CaO as a filler is to stop the process of spontaneous waste heating and, as a result, to reduce the risk of self-ignition. Moreover, the reaction of materials with CaO is short—not exceeding several minutes and the highest temperatures were reached within 2 min after adding CaO. After the mixing process there was no secondary temperature increase. It can be stated that as the result of the process the spent polymer materials became stabilized.

The results of this study are all the more promising because they brought clear evidence that CaO addition can be used for the hygienization of RDF and UFMSW as it is used for the hygienization of sewage sludge.

## Figures and Tables

**Table 1:** Incubation parameters of the analyzed groups of microorganisms.

Microorganisms Groups	Nutrient	Temperature of Incubation [°C]	Time of Incubation [h]
Vegetative bacteria	MPA agar, BTL	37	24
Spores bacteria			
Mold fungi	malt extract agar—MEA, BTL	28	120
Actinobacteria	Pochon’s agar, BTL	28	168
*Staphylococcus* spp.	Chapman’s agar, BTL	37	24
*E. coli*	TBX agar, BTL	44	24
*Salmonella* spp.	SS agar, BTL	37	24
*Shigella* spp.			
*E. faecalis*	Slanetz Bartley substrate, BTL	37	48
*C. perfringens*	agar with sulfate and cycloserine SC, BTL	37	24

**Table 2:** List of basic physicochemical characteristics of refuse-derived fuel (RDF) and undersized fraction of municipal solid waste (UFMSW) (control samples, own research).

Parameter	Unit	Variants	
		RDF (Control Sample)	UFMSW (Control Sample)
Moisture content	% *w*/*w*	20.2 ± 1.9	35.5 ± 2.0
Bulk density	kg·m^−3^	141 ± 13	428 ± 33
pH	-	8.31 ± 0.12	7.54 ± 0.09
AT4	mgO_2_·g^−1^d.m	11.2 ± 0.4	26.3 ± 0.7
Ash content	% d.m.	14.8 ± 0.6	43.5 ± 2.7
Total carbon content	% d.m.	48.7 ± 2.6	29.2 ± 5.2
Sulphur content	% d.m.	0.24 ± 0.06	0.68 ± 0.16
Nitrogen content	% d.m.	0.78 ± 0.12	0.89 ± 0.19
Heat of combustion	MJ·kg^−1^ d.m.	22.5 ± 1.3	10.9 ± 0.7
Calorific value	MJ·kg^−1^	17.5 ± 0.8	6.0 ± 0.7
As	mg·kg^−1^ d.m.	10 ± 3	15 ± 3
Ba	mg·kg^−1^ d.m.	150 ± 11	170 ± 29
Cd	mg·kg^−1^ d.m.	2.2 ± 0.6	3.2 ± 0.3
Cr	mg·kg^−1^ d.m.	56.1 ± 16.0	22.6 ± 9.1
Cu	mg·kg^−1^ d.m.	110.2 ± 12.1	371.1 ± 62.6
Zn	mg·kg^−1^ d.m.	990 ± 160	1438 ± 280
Hg	mg·kg^−1^ d.m.	0.2 ± 0.1	0.6 ± 0.1
Chlorides	mg·kg^−1^ d.m.	1400 ± 56	3050 ± 106
Sulphates	mg·kg^−1^ d.m.	2860 ± 202	23660 ± 890

Mean ± standard error of the mean (n = 3).

**Table 3:** Average number (10^2^ CFU·g^−1^ d.m.) of microorganisms in the control samples (control samples, own research).

Groups od Microorganisms	Variants	
	RDF	UFMSW
Vegetative bacteria	350,000	636,418
Spores bacteria	1567	5207
Mold fungi	1687	3933
*Staphylococcus* spp.	17,380	17,355
*E. coli*	601.4	2091.6
*Salmonella* spp.	65	221.9
*E. faecalis*	25	133.6
*C. perfringens*	24.7	10.6

**Table 4:** Impact of CaO additions on the selected physicochemical properties of analyzed materials.

Samples/The Initial Moisture Content	Moisture Content [% *w*/*w*]	pH	AT4 [mgO_2_·g^−1^d.m]
with 1% CaO addition			
RDF/20%	19.9 ± 0.9	10.40 ± 0.12	11.5 ± 0.7
RDF/30%	28.9 ± 2.9	12.19 ± 0.11	12.5 ± 1.1
UFMSW/35%	35.1 ± 2.2	10.17 ± 0.17	26.0 ± 1.2
UFMSW/50%	48.8 ± 3.1	12.22 ± 0.13	28.1 ± 0.8
UFMSW/60%	58.2 ± 2.0	12.19 ± 0.15	27.6 ± 2.6
with 2% CaO addition			
RDF/20%	19.9 ± 1.1	11.80 ± 0.08	11.5 ± 0.9
RDF/30%	29.2 ± 1.7	12.11 ± 0.12	11.7 ± 1.7
UFMSW/35%	34.2 ± 2.6	11.24 ± 0.08	22.2 ± 2.2
UFMSW/50%	49.2 ± 2.4	12.11 ± 0.09	23.1 ± 3.0
UFMSW/60%	56.6 ± 3.1	12.16 ± 0.10	23.0 ± 2.3
with 5% CaO addition			
RDF/20%	19.5 ± 1.2	11.86 ± 0.13	11.3 ± 0.6
RDF/30%	28.2 ± 3.1	12.35 ± 0.09	11.3 ± 0.9
UFMSW/35%	33.2 ± 2.8	12.39 ± 0.10	20.1 ± 1.3
UFMSW/50%	44.1 ± 2.1	12.34 ± 0.12	16.1 ± 2.0
UFMSW/60%	52.9 ± 2.2	12.48 ± 0.11	16.6 ± 2.2

Mean ± standard error of mean (n = 3).

**Table 5:** Average number (·10^2^ CFU·g^−1^ d.m.) of microorganisms in analyzed samples.

Samples/Initial Moisture Content	Vegetative Bacteria	Spores Bacteria	Mold Fungi	*Staphylococcus* spp.	*E. coli*	*Salmonella* spp.	*E. faecalis*	*C. perfringens*
with 1% CaO addition								
RDF/20%	121132 c	614 ab	1999 a	8626 b	0 a	0 a	0.4 a	0.8 a
RDF/30%	300040 c	738 ab	1151 a	11446 b	1.8 a	2.8 a	18.7 a	0.5 a
UFMSW/35%	140284 c	509 ab	1365 a	10319 b	0 a	0 a	0.6 a	0.6 a
UFMSW/50%	254459 c	738 ab	1521 a	11487 b	2.9 a	3.3 a	19.9 a	0 a
UFMSW/60%	206801 c	574 ab	11539 a	9793 a	4.5 a	1.6 a	16.5 a	0 a
with 2% CaO addition								
RDF/20%	32873 b	720 ab	1332 a	1950 a	0 a	0 a	0 a	0.5 a
RDF/30%	22564 b	117 a	167 a	1688 a	5.7 a	0.4 a	1.2 a	2.4 a
UFMSW/35%	52604 b	747 ab	2140 a	1969 a	0 a	0 a	0 a	0.4 a
UFMSW/50%	25993 b	543 ab	150 a	1512 a	0 a	0.4 a	1.1 a	5.4 a
UFMSW/60%	31201 b	108 ab	170 a	1497 a	9.1 a	0.5 a	0.8 a	1.4 a
with 5% CaO addition								
RDF/20%	32873 b	720 ab	1332 a	1950 a	0 a	0 a	0 a	0.5 a
RDF/30%	22564 b	117 a	167 a	1688 a	5.7 a	0.4 a	1.2 a	2.4 a
UFMSW/35%	11681 a	814 ab	901 a	2131 a	0 a	0.1 a	0 a	0 a
UFMSW/50%	4136 a	4012 b	1753 a	2203 a	0.9 a	0 a	0 a	0 a
UFMSW/60%	1991 a	123 a	1041 a	1114 a	0 a	0 a	0 a	0 a

Different letters within a column indicate a significant difference at *p* < 0.05 according to Tukey’s test.
